# Patients 40 Years of Age or Older Have Comparable Return to Sport Rates, Reinjury Rates, and 2‐Year Patient‐Reported Outcomes After Medial or Lateral Meniscus Repair Compared With Patients Younger Than 40 Years of Age

**DOI:** 10.1002/ars2.70071

**Published:** 2026-09-06

**Authors:** Alexander E. White, Tyler J. Uppstrom, Akshay K. Raghuram, Thun Itthipanichpong, Samarth V. Menta, Robert B. Baldwin, Shu‐Han Wang, Anil S. Ranawat

**Affiliations:** ^1^ Department of Sports Medicine Hospital for Special Surgery New York New York U.S.A.; ^2^ The Steadman Clinic/Steadman Philippon Research Institute Vail Colorado U.S.A.; ^3^ Department of Orthopaedics Chulalongkorn University and King Chulalongkorn Memorial Hospital Bangkok Thailand; ^4^ Biostatistics Core Hospital for Special Surgery New York New York U.S.A.

## Abstract

**Purpose:**

To compare patient‐reported outcomes, return to sport (RTS), and reinjury rates after meniscus repair in patients over and under the age of 40 years old.

**Methods:**

Meniscus repair cases performed at a single tertiary care center between December 2010 and August 2021 with a minimum 2‐year follow‐up data were reviewed. Patient‐reported outcome measures, RTS, and reinjury data were obtained from an institutional knee registry and prospective surveys. Patients were classified as (1) <40 years old with isolated meniscus repair, (2) ≥40 years old with isolated meniscus repair, (3) <40 years old with meniscus repair and anterior cruciate ligament reconstruction, and (4) ≥40 years old with meniscus repair and anterior cruciate ligament reconstruction.

**Results:**

One hundred thirty patients were included, 91 were <40 years old and 39 were ≥40 years old (56% men). Of the tears, 66.2% (n = 86) were medial, 26.9% (n = 35) were lateral, and 6.9% (n = 9) were both medial and lateral. In patients with concomitant anterior cruciate ligament reconstruction, there were no statistically significant differences in 2‐year International Knee Documentation Committee scores (*P* = .58), RTS (*P* = .80), or meniscus repair failure rates (*P* > .99) between different age groups. In isolated meniscus repairs, there were no statistically significant differences in 2‐year International Knee Documentation Committee scores (*P* = .72), RTS (*P* = .24), or meniscus repair failure rates (*P* = .54) between age groups. At 2‐year follow‐up, over 80% of patients in both age groups achieved the minimum clinically important difference across all patient‐reported outcomes, with no significant differences in minimum clinically important difference achievement between patients younger than 40 years and those aged 40 years or older.

**Conclusions:**

In this study, we found that patients 40 years or older who undergo meniscus repair experience similar 2‐year patient‐reported outcome measures, RTS, and failure rates compared with patients younger than 40.

**Level of Evidence:**

Level III, retrospective comparative study.

## Graphical abstract

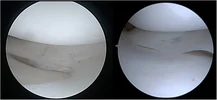

Meniscal injuries, a prevalent pathology often necessitating surgical intervention by orthopaedic surgeons, significantly impact knee joint biomechanics. The menisci play a crucial role in stabilizing the femorotibial joint, distributing axial load, and contributing to proprioception and lubrication of the knee. Damage to or removal of the meniscus can lead to degenerative changes in the tibiofemoral joint. With the advent of innovative techniques and devices, the indications for meniscus repair have expanded.^1^ Meniscal repairs have shown superior postoperative outcomes and long‐term prognoses compared with meniscectomies.^2^ Traditionally, meniscus repair has been favored for younger, more active patients, whereas partial meniscectomy has been preferred for older, less active individuals.^3^ However, recent systematic reviews have shown that patients over 40 years of age undergoing meniscal repair exhibit failure rates and functional outcomes comparable to those reported in younger populations.^4^, ^5^

Age has historically been used as a surrogate marker for biological healing potential, tissue quality, and activity demands when determining whether a meniscal tear is suitable for repair or better treated with partial meniscectomy. Older patients are more likely to present with degenerative tear patterns, reduced meniscal vascularity and cellularity, and higher rates of chondral changes, all factors traditionally believed to compromise postoperative healing. In addition, older individuals may have different functional goals and activity profiles, leading some surgeons to favor partial meniscectomy for more rapid symptom relief. As a result, age cutoffs around 40 years have frequently been used in clinical decision‐making and in the meniscal repair literature, because this threshold aligns with the fourth‐decade decline in intrinsic vascular supply, increasing osteoarthritis risk and decreasing activity levels.^6^, ^7^, ^8^, ^9^, ^10^, ^11^ Recent studies have found no association between increased age and higher failure rates of meniscal repairs,^12^, ^13^ though to date, only a limited number of studies have compared functional outcomes after meniscal repair in age groups above and below 40 years.^14^, ^15^ Although several investigations have identified anterior cruciate ligament (ACLR) as an independent protective factor for meniscal repair, a 2022 systematic review and meta‐analysis by Nepple et al. challenged this notion, showing similar failure rates after meniscus repair, regardless of concomitant ACLR.^12^, ^16^, ^17^ However, this study did not stratify patients >40 years of age.

The purpose of this study was to compare patient‐reported outcomes, return to sport (RTS), and reinjury rates after meniscus repair in patients over and under the age of 40 years old. We hypothesized that patients >40 years of age at the time of meniscal repair with or without concomitant ACLR will have noninferior outcomes for patient‐reported outcome measures (PROMs), RTS rate, and meniscal repair failure rates as compared with those <40 years of age at the time of surgery.

## METHODS

This study received approval from the Institutional Review Board (IRB# 2022‐1243). A retrospective chart review was conducted to analyze patients aged >40 years versus those <40 years who underwent meniscus repair between 2009 and 2022 with at least 2 years of follow‐up data available. Inclusion criteria comprised age >18 years at surgery, isolated medial or lateral meniscal repair or meniscal repair with concomitant ACLR, complete baseline scores for International Knee Documentation Committee (IKDC), Marx scale, and Single Assessment Numeric Evaluation (SANE) in the anterior cruciate ligament (ACL) registry, and complete intraoperative data. It is important to note that patients with 2‐year PROMs data were selected; data on revision meniscus surgery was not required for inclusion. Patients were excluded if they were <18 years old, underwent revision ACLR, meniscal root repairs, lateral extra‐articular augmentation procedures, had prior meniscal surgery, underwent concomitant ligament reconstruction other than ACL, additional cartilage procedures, or knee osteotomy. We stratified patients undergoing isolated meniscus repair and those undergoing meniscus repair with concomitant ACLR, analyzing them separately to evaluate failure rates based on age and concomitant ACLR. Four cohorts were analyzed: isolated meniscal repair in patients >40 years, meniscal repair with concomitant ACLR in patients >40 years, isolated meniscal repair in patients <40 years, and meniscal repair with concomitant ACLR in patients <40 years. Patients who sustained a meniscal tear that was deemed repairable intraoperatively with or without a concomitant ACL tear were indicated for surgery (Figure 1). Tears that occurred in the red‐red zone or red‐white zones were repaired. Tears in the white‐white zone were only repaired in very select cases where tissue quality was felt to be excellent, and partial meniscectomy would have resulted in the removal of >70% of the meniscus.^18^ Techniques employed included all‐inside, inside‐out, inside‐in, all‐inside and inside‐out, all‐inside, and outside‐in. This represents a multisurgeon cohort; however, marrow venting is routinely performed for isolated meniscal repairs, and this standard practice can be explicitly noted. Postoperative rehabilitation protocols varied given the involvement of multiple surgeons and heterogeneous tear patterns but generally included restricted weight‐bearing with the knee locked in extension during ambulation for the first 1 to 2 postoperative weeks.

**FIGURE 1:**
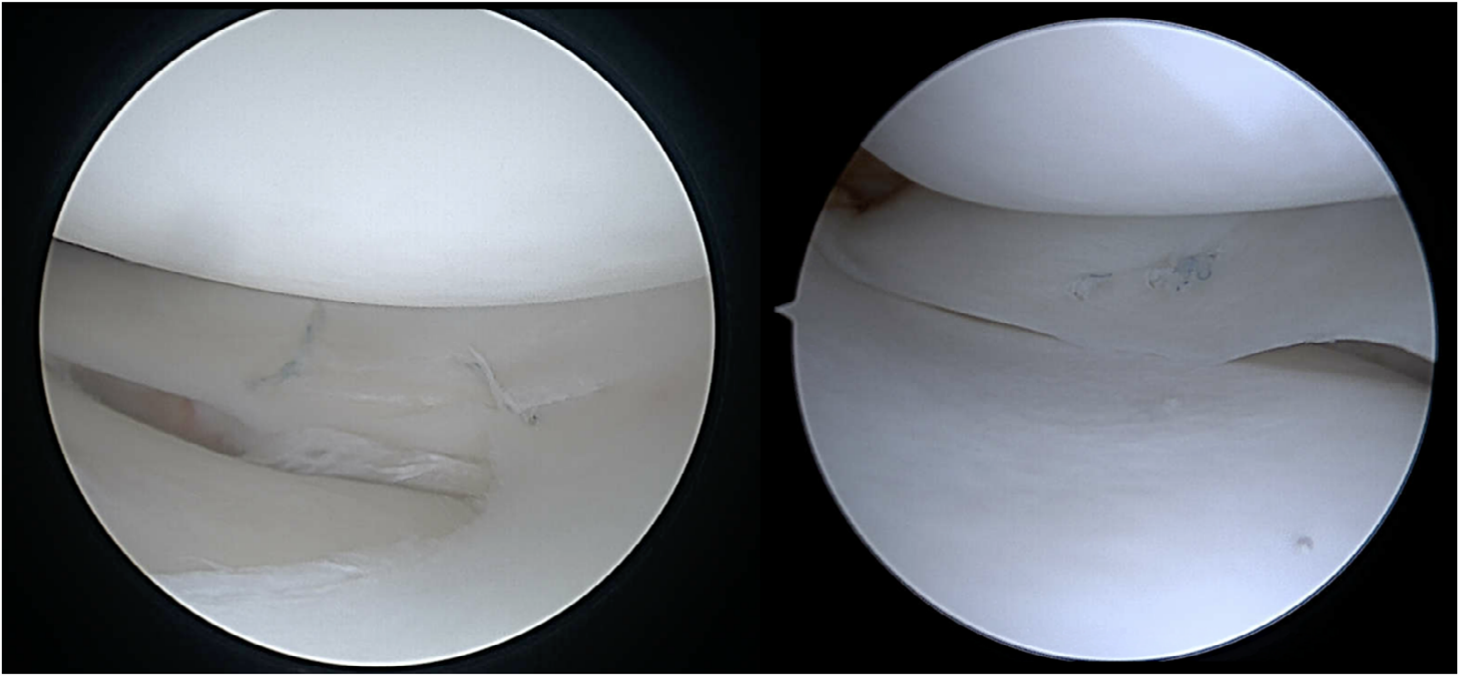
Images that are representative arthroscopic photographs of all‐inside lateral (left) and medial (right) meniscus repairs performed at our institution.

Data were collected from our institution's ACL Registry and Knee Registry, which comprised 2 separate time periods (2009‐2013 and 2019‐2022) to identify patients who underwent meniscal repair with or without concomitant ACLR. There was no available data from 2013 to 2019 because of registry and electronic health record changes at the hospital. Primary outcome measures included PROMs: IKDC Subjective Knee Evaluation, Marx Activity Scale, SANE score, patient‐reported outcomes measurement information system ‐ mobility (PROMIS‐MO), and patient‐reported outcomes measurement information system ‐ pain interference (PROMIS‐PI). These were administered as part of standard clinical care through the electronic medical record at the preoperative visit and at routine postoperative intervals of 1, 2, and 5 years. Secondary outcomes were collected via telephone survey and chart review, including reoperations, RTS, graft rerupture (for ACLR patients), overall satisfaction, meniscal tear characteristics, repair technique, cartilage grade, postoperative complications, range of motion, magnetic resonance imaging evidence of failure, and time to failure. RTS was reported in a binary fashion and was defined as a patient's ability to return to their preoperative sport at the same preinjury level. Patients were matched based on meniscal profiles (based on tear type, location, and zone) to ensure similarity between age groups.

### Data Analysis

Descriptive statistics were calculated using means and standard deviations for continuous variables and frequencies and percentages for discrete variables. Comparative differences between study groups were analyzed with independent sample t‐tests for continuous variables and *χ* ^2^ or Fisher's exact test for discrete variables. Nonparametric equivalents were employed for variables not meeting normality assumptions according to the Shapiro‐Wilk test. Multivariable analyses were performed to evaluate differences between knees undergoing ACLR with or without meniscal repair with respect to PROM scores, meniscal reoperation rates, and return to play. These analyses included demographic variables (i.e., body mass index, sports participation, and ethnicity). Stepwise, multivariable regression models were developed to identify potential risk factors for subsequent meniscus surgery or any subsequent knee procedure while adjusting for confounding variables. Significance was set at *P* < .05, and all tests were 2‐sided. Minimum clinically important difference (MCID) thresholds were calculated using a distribution‐based method because of the absence of validated anchor questions in the registry dataset.^19^ MCID was defined as one‐half of the standard deviation (0.5 standard deviation) of the change (delta) in each PROM, consistent with recommended methodology when anchor‐based approaches are not feasible. The resulting MCID thresholds were 11.7 points for the SANE, 8.4 points for the IKDC, 3.4 points for PROMIS‐PI, and 3.3 points for PROMIS‐MO. MCID thresholds were calculated separately for each outcome using patients with available paired baseline and 2‐year follow‐up data. MCID achievement was assessed at the individual patient level and reported as the proportion (%) of patients meeting or exceeding the MCID threshold for each outcome. Outcomes assessed included the SANE, IKDC, PROMIS‐PI, and PROMIS‐MO. Improvement was defined as an increase in score for SANE, IKDC, and PROMIS‐MO and a decrease in score for PROMIS‐PI. Comparisons of MCID achievement rates between age groups (<40 vs ≥40 years) were performed using *χ* ^2^ testing, with statistical significance set at *P* < .05. This comprehensive statistical approach allowed for a thorough evaluation of meniscal repair outcomes in patients above and below 40 years of age, with potential implications for expanding indications for meniscal repair in older patient populations. All statistical analyses were performed using R v4.3.2 (R Foundation for Statistical Computing, Vienna, Austria).

## RESULTS

There were 130 patients (56% men) included in the final analysis. The mean patient age was 33.6 years old (±10.65), and the mean body mass index was 25.19 (±4.18). Of these patients, 91 (70%) were less than 40 years old and 39 (30%) were greater than or equal to 40 years old. There were 86 patients (66.2%) who underwent medial meniscus repair, 35 patients (26.9%) who underwent lateral meniscus repair, and 9 patients (6.92%) who underwent both medial and lateral meniscus repairs. One hundred nine (83.9%) of the meniscus tears were described as vertical, 7 (5.4%) were bucket handle tears, and 14 patients (10.8%) had both vertical and bucket handle tears. The tear location was at the posterior horn in 85 patients (66.4%), the posterior horn and body in 31 patients (24.2%), posterior horn, body, and anterior horn in 5 patients (3.9%), anterior horn and body in 3 patients (2.3%), anterior horn in 2 patients (1.6%), and meniscal body in 2 patients (1.6%). The tear zone was classified as the red‐red zone in 66 patients (51.2%), red‐white zone in 55 patients (42.6%), red‐red and red‐white zones in 4 patients (3.1%), red‐red, red‐white, and white‐white zones in 3 patients (2.3%), and white‐white zone in 1 patient (0.8%). Of the meniscal repair techniques used, 118 (90.8%) were all‐inside, 5 (3.9%) were all‐inside plus outside‐in, 4 (3.1%) were inside‐out, 2 (1.5%) were all‐inside and inside‐out, and 1 was outside‐in (0.8%) (Table 1).

**TABLE 1:** Baseline Patient Demographics

		
**Variable**	**Mean**	**SD**
Age	33.63	10.65
BMI	25.19	4.18
Sex	*N*	*%*
Male	73	56.15%
Female	57	43.85%
Race	*N*	*%*
White	8	6.15%
African American	96	73.85%
Asian/Pacific Islander	6	4.62%
Other	20	15.38%
Procedure	*N*	*%*
Medial meniscus repair	86	66.15%
Lateral meniscus repair	35	26.92%
Medial and lateral meniscus repair	9	6.92%
Tear type	*N*	*%*
Vertical	109	83.85%
Bucket handle	7	5.38%
Both	14	10.77%
Tear location	*N*	*%*
Posterior horn	85	66.41%
Body	2	1.56%
Anterior horn	2	1.56%
Posterior horn and body	31	24.22%
Anterior horn and body	3	2.34%
Posterior horn, body, and anterior horn	5	3.91%
Tear zone	*N*	*%*
Red‐red	66	51.16%
Red‐white	55	42.64%
White‐white	1	0.78%
Red‐red and red‐white	4	3.10%
Red‐red, red‐white, and white‐white	3	2.33%
Technique	*N*	*%*
All‐inside	118	90.77%
Inside‐out	4	3.08%
Outside‐in	1	0.77%
All‐inside and inside‐out	2	1.54%
All‐inside and outside‐in	5	3.85%

BMI, body mass index; SD, standard deviation.

In patients who underwent a meniscus repair with a concomitant ACLR (N = 99), baseline characteristics were mostly comparable between the <40 and ≥40 groups (Table 2).

**TABLE 2:** Baseline Characteristics for Patients Who Underwent Meniscus Repair With ACL Reconstruction Separated by Age Group

**Variable**	**<40 Years Group**		≥ **40 Years Group**		***P*** **Value**
	**N = 70 (70.71%)**		**N = 29 (29.29%)**		
	**Mean**	**SD**	**Mean**	**SD**	
Age	27.51	5.64	46.79	5.23	
BMI	25.68	4.25	25.49	4.91	.67
Sex					.66
Male	37	52.86%	17	58.62%	
Female	33	47.14%	12	41.38%	
Race					.34
White	3	4.29%	4	13.79%	
African American	50	71.43%	21	72.41%	
Asian/Pacific Islander	5	7.14%	0	0	
Other	12	17.14%	4	13.80%	
Laterality					.51
Right	35	50%	17	58.62%	
Left	35	50%	12	41.38%	
Procedure					>.99
Medial meniscus repair	44	62.86%	19	65.52%	
Lateral meniscus repair	19	27.14%	8	27.59%	
Medial and lateral meniscus repair	7	10.00%	2	6.90%	
Tear type					.42
Vertical	61	87.14%	25	86.21%	
Bucket handle	6	8.57%	1	3.45%	
Both	3	4.29%	3	0.34%	
Tear location					.83
Posterior horn	48	69.57%	19	67.86%	
Body	1	1.45%	1	3.57%	
Anterior horn	2	2.90	0	0	
Posterior horn and body	15	21.74%	7	25.00%	
Anterior horn and body	1	1.45%	1	3.57%	
Posterior horn, body and anterior horn	2	2.90%	0	0	
Tear zone					.24
Red‐red	32	45.71%	18	64.29%	
Red‐white	32	45.71%	8	28.57%	
White‐white	1	1.43%	0	0	
Red‐red and red‐white	2	2.86%	2	7.14%	
Red‐red, red‐white and white‐white	3	4.29%	0	0	
Technique					.92
All‐inside	64	91.43%	27	93.10%	
Inside‐out	2	2.86%	1	3.45%	
Outside‐in	1	1.43%	0	0	
All‐inside and inside‐out	2	2.86%	0	0	
All‐inside and outside‐in	1	1.43%	1	3.45%	
Medial plateau cartilage grade					.07
0	62	88.57%	21	72.41%	
1	7	10.00%	7	24.14%	
2	0	0	1	3.45%	
3	1	1.43%	0	0	
Medial femoral condyle cartilage grade					.10
0	57	81.43%	20	68.97%	
1	9	12.86%	3	10.34%	
2	2	2.86%	4	13.79%	
3	1	1.43%	2	6.90%	
4	1	1.43%	0	0	
Lateral plateau cartilage grade					**.007**
0	60	85.71%	18	62.07%	
1	8	11.43%	10	34.48%	
2	2	2.86%	0	0	
3	0	0	1	3.45%	
Lateral femoral condyle cartilage grade					**.026**
0	61	87.14%	20	68.97%	
1	9	12.86%	7	24.14%	
2	0	0	2	6.90%	
3	0	0	0	0	

*Note*: Bold values are considered statistically significant at a *P* value threshold of <.05.

ACL, anterior cruciate ligament; BMI, body mass index; SD, standard deviation.

The ≥40 group had a significantly higher incidence of chondral wear in the lateral femoral condyle (*P* = .026) and lateral tibial plateau (*P* = .007) compared with the <40 group (Table 2). In this group of patients, 2‐year IKDC scores were 85.91 in the <40 group and 84.23 in the ≥40 group (*P* = .58). Additionally, Marx scores at the 2‐year postoperative time point were 9.01 for the <40 group and 7.55 for the ≥40 group, which was a statistically significant difference (*P* < .001) (Table 3). RTS rates were 70% (47/67) for the <40 group and 75% (21/28) for ≥40 (*P* = .80). Failure of meniscus repair as determined by revision meniscus surgery was 20.0% (14/70) for the <40 group and 20.7% (6/29) for the ≥40 group (*P* > .99). The overall failure rate in patients who underwent meniscus repair in the setting of an ACLR was 20.2% (20/99), at an average follow‐up of 7.6 years (range 3.9‐13.2 years).

**TABLE 3:** PROMs for Patients Who Underwent Meniscus Repair With ACL Reconstruction Separated by Age Group

**Variable**	**<40 Years Group**		**40 Years Group**	**SD**	***P*** **Value**
	**N = 70 (70.71%)**		**N = 29 (29.29%)**		
	**Mean**	**SD**	**Mean**		
SANE score	82.19	15.08	80.14	22.08	.72
IKDC: baseline	46.45	16.20	44.53	16.73	.48
IKDC: 2 years	85.91	12.84	84.23	13.75	.58
Marx: baseline	11.99	4.19	10.34	5.49	.15
Marx: 2 years	9.01	4.55	7.55	5.66	** <.001**

*Note*: Bold values are considered statistically significant at a *P* value threshold of <.05.

ACL, anterior cruciate ligament; IKDC, International Knee Documentation Committee; PROMs, patient‐reported outcome measures; SANE, Single Assessment Numeric Evaluation; SD, standard deviation.

In patients who underwent an isolated meniscus repair (N = 31), baseline characteristics between the 2 groups were similar (Table 4).

**TABLE 4:** Baseline Characteristics for Patients Who Underwent Isolated Meniscus Repair Separated by Age Group

**Variable**	** <40 Years Group**		≥ **40 Years Group**		***P*** **Value**
	**N = 21 (67.74%)**		**N = 10 (32.26%)**		
	**Mean**	**SD**	**Mean**	**SD**	
Age	28.67	5.73	48.7	7.97	
BMI	23.92	3.34	23.36	1.69	>.99
Sex					.13
Male	15	71.43%	4	40.00%	
Female	6	28.57%	6	60.00%	
Race					.90
White	1	4.76%	0	0%	
African American	16	76.19%	9	90.00%	
Asian/Pacific Islander	1	4.76%	0	0%	
Other	3	14.28%	1	10.00%	
Laterality					>.99
Right	9	42.86%	4	40.00%	
Left	12	57.14%	6	60.00%	
Procedure					.38
Medial meniscus repair	17	80.59%	6	60%	
Lateral meniscus repair	4	19.05%	4	40%	
Medial and lateral meniscus repair	0	0	0	0	
Tear type					>.99
Vertical	15	71.43%	8	80.00%	
Bucket handle	0	0%	0	0%	
Both	6	28.57%	2	20.00%	
Tear location					.82
Posterior horn	13	61.90%	5	50.00%	
Body	0	0	0	0	
Anterior horn	0	0	0	0	
Posterior horn and body	5	23.81%	4	40%	
Anterior horn and body	1	4.76%	0	0	
Posterior horn, body and anterior horn	2	9.52%	1	10%	
Tear zone					.46
Red‐red	12	57.14%	4	40%	
Red‐white	9	42.86%	6	60%	
White‐white	0	0	0	0	
Red‐red and red‐white	0	0	0	0	
Red‐red, red‐white and white‐white	0	0	0	0	
Technique					.68
All‐inside	17	80.59%	10	100%	
Inside‐out	1	4.76%	0	0	
Outside‐in	0	0	0	0	
All‐inside and inside‐out	0	0	0	0	
All‐inside and outside‐in	3	14.29%	0	0	
Medial plateau cartilage grade					.69
0	18	85.71%	10	100%	
1	2	8.52%	0	0	
2	1	4.76%	0	0	
3	0	0	0	0	
Medial femoral condyle cartilage grade					.08
0	18	85.71%	8	80.00%	
1	3	14.29%	0	0	
2	0	0	1	10.00%	
3	0	0	1	10.00%	
4	0	0	0	0	
Lateral plateau cartilage grade					>.99
0	19	90.48%	9	90.00%	
1	1	4.76%	0	0	
2	1	4.76%	1	10%	
3	0	0	0	0	
Lateral femoral condyle cartilage grade					>.99
0	18	85.71%	10	100.00%	
1	1	4.76%	0	0	
2	1	4.76%	0	0	
3	1	4.76%	0	0	

BMI, body mass index; SD, standard deviation.

In this group of patients, 2‐year IKDC scores were 81.67 for the <40 group and 83.68 for the ≥40 group (*P* = .72). SANE scores at 2‐years postoperatively were 78.81 in the <40 group and 73.40 in the ≥40 group (*P* > .99). Marx scores at the 2‐year postoperative time point were 7.95 for the <40 group and 5.5 for the ≥40 group, which was a statistically significant difference (*P* < .001) (Table 5). RTS rates were 50% (10/20) for the <40 group and 80% (8/10) for ≥40 (*P* = .24). Failure of meniscus repair as determined by revision meniscus surgery was 11.1% (2/18) for the <40 group and 0.0% (0/9) for the ≥40 group (*P* = .54). The overall failure rate in patients who underwent isolated meniscus repair was 7.4% (2/27), at an average follow‐up of 4.0 years (range 2.6‐11.1 years).

**TABLE 5:** PROMs for Patients Who Underwent Isolated Meniscus Repair Separated by Age Group

**Variable**	** <40 Years Group**		**40 Years Group**	**SD**	***P*** **Value**
	**N = 21 (67.74%)**		**N = 10 (32.26%)**		
	**Mean**	**SD**	**Mean**		
SANE score	78.81	16.39	73.4	26.37	>.99
IKDC: baseline	50.58	18.26	42.34	18.59	.26
IKDC: 2 years	81.67	15.18	83.68	13.92	.72
Marx: baseline	10.29	4.74	10.3	2.75	.64
Marx: 2 years	7.95	4.55	5.5	4.01	**<.001**

*Note*: Bold values are considered statistically significant at a *P* value threshold of <.05.

IKDC, International Knee Documentation Committee; PROMs, patient‐reported outcome measures; SANE, Single Assessment Numeric Evaluation; SD, standard deviation.

At 2‐year follow‐up, a high proportion of patients in both age groups achieved clinically meaningful improvement across all outcome measures (Table 6). Among patients younger than 40 years, MCID was achieved by 88.7% for SANE, 92.2% for IKDC, 85.2% for PROMIS‐PI, and 83.3% for PROMIS‐MO. Among patients aged 40 years or older, MCID was achieved by 96.2% for SANE, 98.1% for IKDC, 81.8% for PROMIS‐PI, and 93.3% for PROMIS‐MO.

**TABLE 6:** MCID Thresholds and Proportion of Patients Achieving MCID by Age Group

**Outcome Measure**	**MCID Threshold**	** <40 Years: % Achieved MCID**	** ≥40 Years: % Achieved MCID**	***P*** **Value**
SANE	11.7	88.7%	96.2%	.44
IKDC	8.4	92.2%	98.1%	.19
PROMIS‐PI	3.4	85.2%	81.8%	.74
PROMIS‐MO	3.3	83.3%	93.3%	.66

IKDC, International Knee Documentation Committee; MCID, minimum clinically important difference; PROMIS‐MO, patient‐reported outcomes measurement information system ‐ mobility; PROMIS‐PI, patient‐reported outcomes measurement information system ‐ pain interference; SANE, Single Assessment Numeric Evaluation.

There were no statistically significant differences in the proportion of patients achieving MCID between age groups for any outcome measure (SANE, *P* = .44; IKDC, *P* = .19; PROMIS‐PI, *P* = .74; PROMIS‐MO, *P* = .66), indicating comparable rates of clinically meaningful improvement in patients younger and older than 40 years.

## DISCUSSION

In this study, we found that meniscus repair can be performed successfully in patients over the age of 40 who have an amenable tear type. Meniscus repair was performed almost exclusively in patients with good tissue quality (no plastic deformation) who have tears that occur in the red‐red or red‐white zones. There were rare exceptions where tears in the white‐white zone were repaired because a partial meniscectomy would have resulted in greater than 70% of the meniscus being removed. There were no differences in RTS rates or meniscus repair failure rates when comparing patients who were younger than 40 to those who were 40 or older. Although Marx activity scores were significantly lower for the 40 or older group, the remainder of PROMs did not differ between age groups postoperatively. All of these findings are upheld in the setting of isolated meniscus repair and meniscus repair during concomitant ACLR. This study challenges the typical practice that favors partial meniscectomy in patients who are over the age of 40 and suggests that meniscus repair can be successfully performed in patients 40 years or older who have a tear pattern that is amenable to repair.

This study used all available cases that met the inclusion/exclusion criteria of this retrospective series. A post hoc power analysis suggests that larger sample sizes would be necessary to find these observed differences as statistically significant. In our study, the differences observed between patients under 40 and those 40 and older after medial meniscus surgery were smaller than previously published values for the MCID and minimal detectable change for the outcomes assessed.^20^, ^21^ Future studies should consider equivalence or noninferiority designs with larger sample sizes to more definitively assess whether outcomes are truly comparable between age groups.

Steadman et al. reported results of their inside‐out meniscus repair in 339 patients and found that failure rates—defined as meniscus reoperation—did not differ between those younger than 40 and those 40 or older.^14^ The findings of the present study support those reported by Steadman and colleagues with no discernable differences noted in failure rates when comparing age groups. It is important to note, however, that 90% of patients in the present study underwent all‐inside suture repair, whereas Steadman et al. used a traditional inside‐out repair technique. Similarly, in a systematic review of the literature performed in 2018, Everhart et al. compared functional outcomes in 61 patients >40 years old and 164 patients <40 years old and found no significant difference in failure rates, but their analysis did not stratify outcomes based on concomitant ACLR, which is a common and potentially important modifier of meniscal healing.^4^ Meniscal repairs are frequently performed in conjunction with ACLR, with meniscal pathology reported in more than half of ACL injuries.^22^ Few, if any, studies, however, have separated this population of patients based on their exposure to concomitant ACLR surgery. The present study addresses this gap by separately analyzing isolated repairs and repairs performed with ACLR in cohorts above and below 40 years of age. Specifically, patients were separated based on their exposure to a concomitant ACLR, and it was found that there was no significant difference in meniscus repair failure rates between age groups. For both the under‐40 group and the 40‐or‐over group, the rate of meniscus failure was 20% when the patient underwent a concomitant ACLR. In patients who underwent an isolated meniscus repair, the failure rate was 11% in the less than 40 group and 0% in the 40 or older group. These differences (11% vs 0%) in the isolated meniscus repair group were not statistically significant. Although significant differences were not noted between age groups, it is interesting to note that the patients who underwent concomitant ACLR had a higher overall failure rate (20%) compared with the isolated meniscus repair group (7.4%). This finding is of interest given the mixed literature comparing meniscus repair success when performed at the time of ACLR compared with isolated meniscus repairs.^23^, ^24^, ^25^ Therefore, the present study provides further evidence that meniscus repair can be successfully performed in patients over the age of 40 regardless of the presence of a concomitant ACLR surgery. Additionally, although not the primary intention of this study, the findings suggest that isolated all‐inside meniscus repair may fail less frequently than meniscus repairs performed at the time of ACLR. Future high‐level studies, however, are needed to further elucidate this finding.

IKDC and SANE scores similarly did not differ significantly postoperatively between the 2 age groups at the 2‐year time point. Of note, when comparing Marx scores between the 2 age groups, the 40 or older group reported significantly lower 2‐year scores. Given the equivalence of the other measured PROMs, however, the difference in Marx scores may be a lower activity level that is confounded by the fact that this is an overall older patient population. Baseline Marx scores in the 40 years or older group were 1.5 points lower in the concomitant ACLR group, and although this baseline difference was not statistically significant, it is the same margin of difference between the groups identified postoperatively. In contrast, however, the isolated meniscus repair group had similar baseline Marx scores between age groups, but significantly different postoperative Marx scores. Therefore, it should be acknowledged that although other PROMs were equivalent between age groups after meniscus repair, the Marx score, which characterizes patient activity level, was lower in patients 40 years or older. Similarly, Steadman et al. found most PROMs to be equivalent between age groups after meniscus repair; however, they reported a significantly lower Tegner activity scale score in the 40 and older age group.^14^ Taken together, the results of the present study and those reported by Steadman et al. may be explained by the confounder of age that is inherently associated with lower activity levels. Although this is an important finding to note, it should be considered in the greater context of the study, which suggests overall success of meniscus repair for patients 40 years or older.

In addition to comparing postoperative PROM scores between age groups, patients showed similarly high rates of clinically meaningful improvement as assessed by MCID. More than 80% of patients in both age groups achieved MCID across all evaluated outcome measures, and no significant differences in MCID achievement were observed between patients younger than 40 years and those aged 40 years or older. These findings suggest that older patients not only achieve similar average outcome scores but also experience comparable rates of patient‐perceived clinical benefit after meniscus repair. This further supports meniscal preservation strategies in appropriately selected patients regardless of chronological age. It is important to note that MCID analysis was not performed for the Marx Activity Rating Scale, because MCID methodology applies to continuous measures in which changes in score reflect patient‐perceived clinical improvement. The Marx scale reflects activity frequency rather than symptom burden or functional health status, and changes in score may reflect lifestyle or activity choice rather than treatment‐related benefit. Therefore, the Marx scale was excluded from MCID analyses.

Despite purported concerns regarding the recovery capacity after meniscus repair in patients 40 years or older, the data of the present study show no significant difference in RTS rates comparing this age group to younger patients. In fact, although the differences were not significant, the 40 and older age group returned to sport at a higher rate in both the concomitant ACLR cohort (75% vs 70%, *P* = .80) and in the isolated meniscus repair cohort (80% vs 50%, *P* = .24). These findings are important and should push surgeons away from using strict age criteria for determining repairability of meniscus tears in patients who desire to return to sporting activity. Although younger patients may have a more robust biological healing response and a desire to return to higher level activities, the present study highlights that this 40 and older age group can successfully return to their desired activities after meniscus repair.

### Limitations

The present study must be considered in the context of its limitations. Despite best efforts to control for confounding variables, there are likely influencing patient factors that are not captured as a result of the study design. The minimum 2‐year follow‐up for this study is relatively short, and results may differ with a longer period of follow‐up. Additionally, because data on revision meniscus surgery was not required for inclusion, a fewer number of patients were analyzed for meniscus repair failure. Certain secondary outcomes, including RTS, were collected retrospectively and were therefore subject to incomplete response and attrition; analyses of RTS were limited to patients with available data, and the results should be interpreted in the context of potential response bias. Timing to RTS was not reported in this study, which may bias the results of the RTS data such that older patients return at a higher rate because they are doing so at a later time point. This study was conducted at a single institution, and therefore, the results may not be generalizable to the greater population. We were not able to disaggregate data by sex because we used a convenience sample. In accordance with sex and gender equity in research guidelines, we acknowledge that data were not disaggregated by sex. This analysis was not performed because the study data had been fully analyzed prior to manuscript preparation and retrospective disaggregation was not feasible given the dataset structure. Furthermore, sex‐stratified subgroup analyses would have substantially reduced statistical power given the overall sample size. Our institution did not record patient data within a registry during 2013 to 2019; consequently, patient data from 2009 to 2013 and 2019 to 2022 were analyzed. Given the small number of patients in the isolated meniscus repair cohort aged 40 years or older (n = 9) and the observed 0% failure rate, the possibility of type II error should be considered. The addition of even 1 or 2 failures in this group would have resulted in a 10% to 20% failure rate, underscoring the sensitivity of our findings to small changes in outcome events. Furthermore, given the retrospective nature of this study, selection bias may have played a role in skewing the results of this study and should be considered when interpreting the findings.

## CONCLUSIONS

In this study, we found that patients 40 years or older who undergo meniscus repair experience similar 2‐year PROMs, RTS, and failure rates compared with patients younger than 40.

## DISCLOSURES

The authors (A.E.W., T.J.U., A.K.R., T.I., S.V.M., R.B.B., S‐H.W., A.S.R.) declare that they have no known competing financial interests or personal relationships that could have appeared to influence the work reported in this article.

## References

[1] Karia M , Ghaly Y , Al‐Hadithy N , Mordecai S , Gupte C . Current concepts in the techniques, indications and outcomes of meniscal repairs. Eur J Orthop Surg Traumatol. 2019;29:509‐520. 10.1007/s00590-018-2317-5 PMC6423358 30374643

[2] Lee WQ , Gan JZ , Lie DTT . Save the meniscus ‐ Clinical outcomes of meniscectomy versus meniscal repair. J Orthop Surg. 2019;27:2309499019849813. 10.1177/2309499019849813 31117923

[3] Heckmann TP , Barber‐Westin SD , Noyes FR . Meniscal repair and transplantation: Indications, techniques, rehabilitation, and clinical outcome. J Orthop Sports Phys Ther. 2006;36:795‐814. 10.2519/jospt.2006.2177 17063840

[4] Everhart JS , Higgins JD , Poland SG , Abouljoud MM , Flanigan DC . Meniscal repair in patients age 40 years and older: A systematic review of 11 studies and 148 patients. Knee. 2018;25:1142‐1150. 10.1016/j.knee.2018.09.009 30414793

[5] Jaibaji R , Khaleel F , Jaibaji M , Volpin A . Outcomes of meniscal repair in patients aged 40 and above: A systematic review. J Clin Med. 2023;12:6922. 10.3390/jcm12216922 PMC10649032 37959387

[6] Metcalf MH , Barrett GR . Prospective evaluation of 1485 meniscal tear patterns in patients with stable knees. Am J Sports Med. 2004;32:675‐680. 10.1177/0095399703258743 15090384

[7] Culvenor AG , Øiestad BE , Hart HF , Stefanik JJ , Guermazi A , Crossley KM . Prevalence of knee osteoarthritis features on magnetic resonance imaging in asymptomatic uninjured adults: A systematic review and meta‐analysis. Br J Sports Med. 2019;53:1268‐1278. 10.1136/bjsports-2018-099257 PMC6837253 29886437

[8] Arnoczky SP , Warren RF . Microvasculature of the human meniscus. Am J Sports Med. 1982;10:90‐95. 10.1177/036354658201000205 7081532

[9] Petersen W , Tillmann B . Age‐related blood and lymph supply of the knee menisci. A cadaver study. Acta Orthop Scand. 1995;66:308‐312. 10.3109/17453679508995550 7676815

[10] Michel PA , Domnick CJ , Raschke MJ , et al. Age‐related changes in the microvascular density of the human meniscus. Am J Sports Med. 2021;49:3544‐3550. 10.1177/03635465211039865 34591716

[11] Duong V , Oo WM , Ding C , Culvenor AG , Hunter DJ . Evaluation and treatment of knee pain: A review. JAMA. 2023;330:1568‐1580. 10.1001/jama.2023.19675 37874571

[12] Lyman S , Hidaka C , Valdez AS , et al. Risk factors for meniscectomy after meniscal repair. Am J Sports Med. 2013;41:2772‐2778. 10.1177/0363546513503444 24036573

[13] Poland S , Everhart JS , Kim W , Axcell K , Magnussen RA , Flanigan DC . Age of 40 years or older does not affect meniscal repair failure risk at 5 years. Arthroscopy. 2019;35:1527‐1532. 10.1016/j.arthro.2018.11.061 31000396

[14] Steadman JR , Matheny LM , Singleton SB , et al. Meniscus suture repair: Minimum 10‐year outcomes in patients younger than 40 years compared with patients 40 and older. Am J Sports Med. 2015;43:2222‐2227. 10.1177/0363546515591260 26187129

[15] Everhart JS , Magnussen RA , Poland S , et al. Meniscus repair 5‐year results are influenced by patient pre‐injury activity level but not age group. Knee. 2020;27:157‐164. 10.1016/j.knee.2019.11.005 31806508

[16] Turcotte JJ , Maley AD , Levermore SB , Petre BM , Redziniak DE . Risk factors for all‐inside meniscal repair failure in isolation and in conjunction with anterior cruciate ligament reconstruction. Knee. 2021;28:9‐16. 10.1016/j.knee.2020.10.012 33278740

[17] Nepple JJ , Block AM , Eisenberg MT , Palumbo NE , Wright RW . Meniscal repair outcomes at greater than 5 years: A systematic review and meta‐analysis. J Bone Joint Surg Am. 2022;104:1311‐1320. 10.2106/JBJS.21.01303 35856932

[18] Bansal S , Floyd ER , Kowalski AM , et al. Meniscal repair: The current state and recent advances in augmentation. J Orthop Res. 2021;39:1368‐1382. 10.1002/jor.25021 PMC8249336 33751642

[19] Watt JA , Veroniki AA , Tricco AC , Straus SE . Using a distribution‐based approach and systematic review methods to derive minimum clinically important differences. BMC Med Res Methodol. 2021;21:41. 10.1186/s12874-021-01228-7 PMC7912575 33637039

[20] Westermann RW , Wright RW , Spindler KP , Huston LJ ; MOON Knee Group , Wolf BR . Meniscal repair with concurrent anterior cruciate ligament reconstruction: Operative success and patient outcomes at 6‐year follow‐up. Am J Sports Med. 2014;42:2184‐2192. 10.1177/0363546514536022 PMC4451057 25023440

[21] Maheshwer B , Wong SE , Polce EM , et al. Establishing the minimal clinically important difference and patient‐acceptable symptomatic state after arthroscopic meniscal repair and associated variables for achievement. Arthroscopy. 2021;37:3479‐3486. 10.1016/j.arthro.2021.04.058 33964390

[22] Smith JP 3rd , Barrett GR . Medial and lateral meniscal tear patterns in anterior cruciate ligament‐deficient knees. A prospective analysis of 575 tears. Am J Sports Med. 2001;29:415‐419. 10.1177/03635465010290040501 11476378

[23] Toman CV , Dunn WR , Spindler KP , et al. Success of meniscal repair at anterior cruciate ligament reconstruction. Am J Sports Med. 2009;37:1111‐1115. 10.1177/0363546509337010 PMC3692358 19465734

[24] Uzun E , Misir A , Kizkapan TB , Ozcamdalli M , Akkurt S , Guney A . Factors affecting the outcomes of arthroscopically repaired traumatic vertical longitudinal medial meniscal tears. Orthop J Sports Med. 2017;5:2325967117712448. 10.1177/2325967117712448 PMC5484431 28680898

[25] Vaquero‐Picado A , Rodríguez‐Merchán EC . Arthroscopic repair of the meniscus: Surgical management and clinical outcomes. EFORT Open Rev. 2018;3:584‐594. 10.1302/2058-5241.3.170059 PMC6275851 30595844

